# Supplementary material for: The Association between the Pan-Immune-Inflammation Value and Cancer Prognosis: A Systematic Review and Meta-Analysis
Source: Cancers (Basel). 2022 May 27;14(11):2675. doi: 10.3390/cancers14112675 (PMC9179577; doi:10.3390/cancers14112675)
Supplement: Supplementary file 1 [file cancers-14-02675-s001.zip › cancers-1694062-supplementary.pdf]

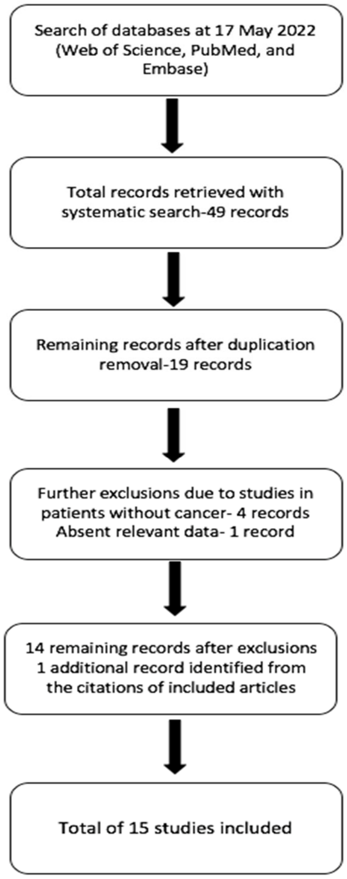

Supplementary Figure S1. PRISMA flow diagram

Study or Subgroup	log[Hazard Ratio]	SE	Weight	Hazard Ratio IV, Random, 95% CI	Year
**1.1.1 All Cohort**					
Fucà 2020	0.6981	0.1261	14.0%	2.01 [1.57, 2.57]	2020
Efil 2021	0.8862	0.2276	8.4%	2.43 [1.55, 3.79]	2021
Guven 2021	0.523	0.241	7.8%	1.69 [1.05, 2.71]	2021
Ligorio 2021	2.2492	0.6427	1.7%	9.48 [2.69, 33.41]	2021
Sahin 2021	0.4943	0.226	8.4%	1.64 [1.05, 2.55]	2021
Zeng 2021	0.8582	0.3587	4.5%	2.36 [1.17, 4.76]	2021
Corti 2021	1.0986	0.3571	4.5%	3.00 [1.49, 6.04]	2021
Fucà 2021	0.7227	0.2349	8.1%	2.06 [1.30, 3.26]	2021
Sato 2022	0.9103	0.2402	7.8%	2.49 [1.55, 3.98]	2022
Gambichler 2022	0.2294	0.4589	3.0%	1.26 [0.51, 3.09]	2022
Lin 2022	0.5423	0.236	8.0%	1.72 [1.08, 2.73]	2022
Baba 2022	0.3148	0.1505	12.4%	1.37 [1.02, 1.84]	2022
Perez-Martelo 2022	0.5988	0.2342	8.1%	1.82 [1.15, 2.88]	2022
Chen 2022	1.5476	0.4359	3.3%	4.70 [2.00, 11.04]	2022
**Subtotal (95% CI)**			**100.0%**	**2.01 [1.70, 2.38]**	
Heterogeneity: Tau² = 0.04; Chi² = 21.63, df = 13 (P = 0.06); I² = 40%					
Test for overall effect: Z = 8.08 (P < 0.00001)					
**Total (95% CI)**			**100.0%**	**2.01 [1.70, 2.38]**	
Heterogeneity: Tau² = 0.04; Chi² = 21.63, df = 13 (P = 0.06); I² = 40%					
Test for overall effect: Z = 8.08 (P < 0.00001)					
Test for subgroup differences: Not applicable					

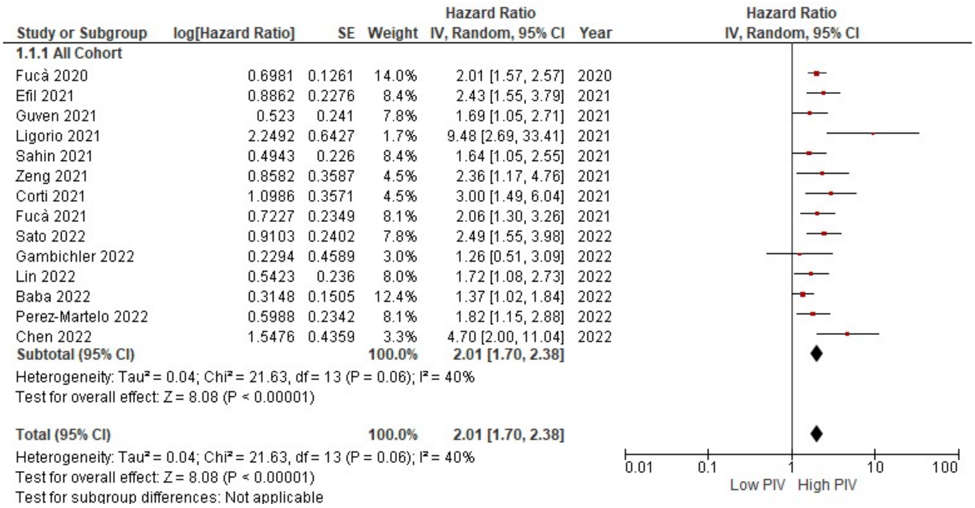

Supplementary Figure S2. Overall survival with a fixed-effect meta-analysis after the exclusion of study by Susok et al.

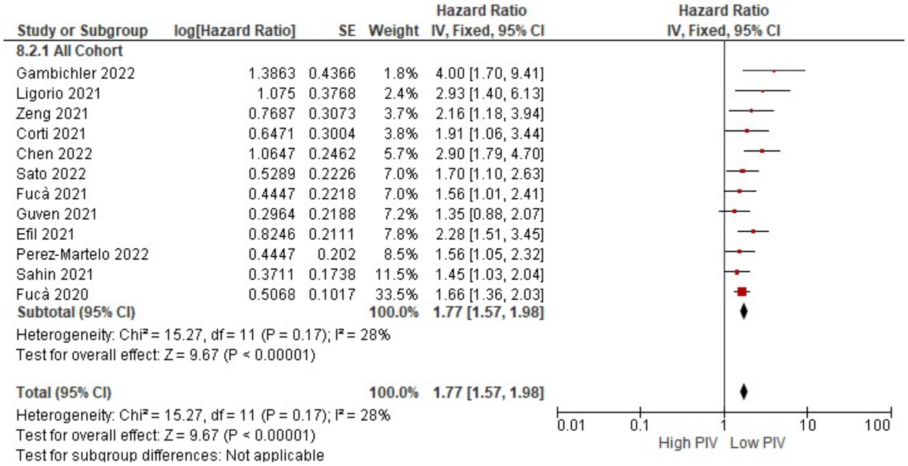

Supplementary Figure S3. Progression-free survival with a fixed-effect meta-analysis after the exclusion of study by Susok et al.

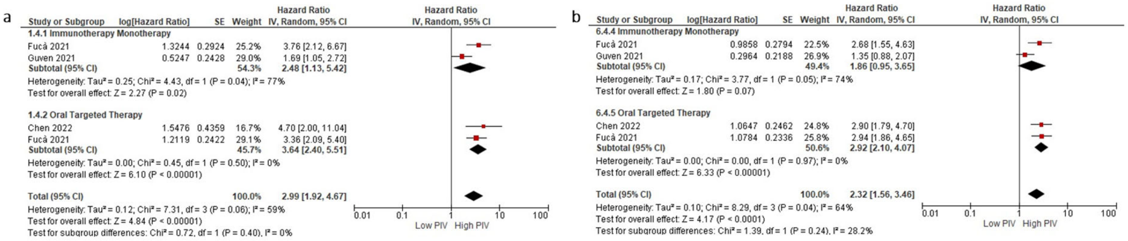

Supplementary Figure S4. Subgroup analyses according to treatment type in OS (a) and DFS/PFS (b). Lines(○) indicate 95% CIs. Diamond(♦) indicates the pooled effect size.
